# Innovative management of intestinal obstruction with colonic transendoscopic enteral tubing

**DOI:** 10.1055/a-2199-4663

**Published:** 2023-11-21

**Authors:** You Yu, Faming Zhang, Bota Cui

**Affiliations:** 1 637622 Department of Microbiota Medicine and Medical Center for Digestive Diseases, The Second Affiliated Hospital of Nanjing Medical University, Nanjing, China; 2 637622 Key Lab of Holistic Integrative Enterology, The Second Affiliated Hospital of Nanjing Medical University, Nanjing, China; 3 Department of Microbiota Medicine, Sir Run Run Hospital, Nanjing Medical University, Nanjing, China

Managing intestinal obstruction in patients with Crohn’s disease (CD) is clinically challenging if emergency surgery is to be avoided 1 . Here, we present a case involving a patient with CD suffering from acute intestinal obstruction who was unresponsive to conservative treatments and successfully avoided emergency surgery through a novel technique for bowel decompression.

A 50-year-old man with a 15-year history of CD was urgently admitted due to abdominal distension, which had worsened over a week. In the preceding 3 months, he had undergone exclusive enteral nutrition. The abdominal distension persisted despite symptomatic measures such as fasting and gastrointestinal decompression. Computed tomography revealed a stenosis, 60 mm in length, at the terminal ileum, which was unsuitable for endoscopic dissection or balloon dilation. A colonoscopy followed a cleansing enema to further evaluate the bowel condition, and colonic transendoscopic enteral tubing (TET) was performed to resolve the obstruction ( Video 1 ).

Management of intestinal obstruction due to a strictured ileocecal valve using colonic transendoscopic enteral tubing.Video 1

Colonic TET is a versatile technology used for managing various conditions in the entire colon, including fecal microbiota transplantation, drug administration, and decompression for endoscopy-associated perforation 2 3 . In this case, colonic TET was innovatively employed to decompress the intestine following obstruction. The TET tube successfully passed through the stenosis that was causing the obstruction ( Fig. 1 ). Decompression was initiated immediately upon the passage of the colonic TET through the stenosis and was well tolerated by the patient. This intervention rapidly alleviated the patient’s abdominal distension, resulting in a flattened abdomen ( Fig. 2 ). A total of 8135 mL of intestinal fluid was drained through the TET tube over 11 days, which created favorable conditions for the subsequent therapy. After sufficient preoperative treatment, the patient underwent successful right colon and terminal ileal resection. Post-surgery, the TET tube remained in the intestine for 1 week for continued drainage and decompression, thus averting post-surgery infection and promoting wound healing.

**Fig. 1:**
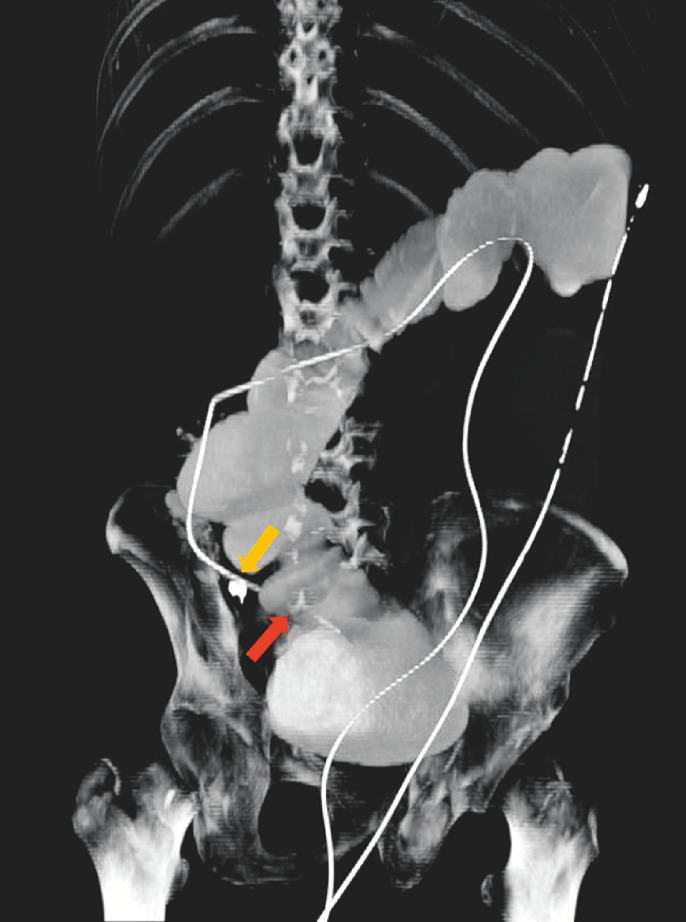
Computed tomography (CT). Approximately 200 mL of contrast agent was injected into the intestine through the colonic transendoscopic enteral tubing (TET) tube. Following a 10-minute ambulation, CT was conducted and displayed the entire TET tube, dilated distal ileum, and a segment of the proximal colon. The yellow arrow indicates clips fixed onto the intestinal wall. The red arrow highlights the TET tube passing through the stricture into the terminal ileum.

**Fig. 2:**
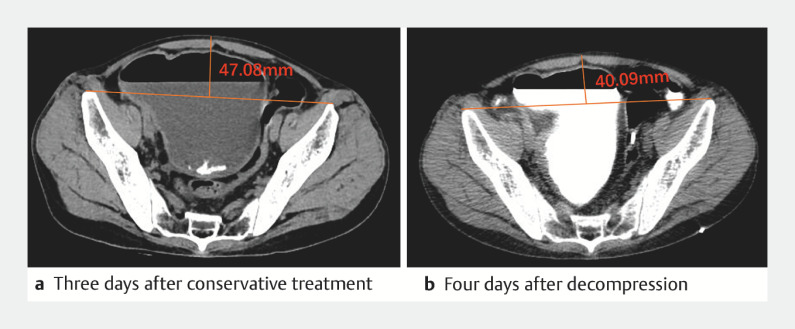
Computed tomography (CT) at the same level before and after decompression, after injecting 200 mL of contrast agent. The measurements show the vertical distance from the connecting line of the highest point of the bony landmarks on both sides to the highest point of the abdomen. **a** The vertical distance was 47.08 mm 3 days after conservative treatment. **b** Over 4 days of decompression using TET, a total of 2800 mL of intestinal fluid was drained and the vertical distance was reduced to 40.09 mm.

Endoscopy_UCTN_Code_TTT_1AQ_2AF

## Notes

Endoscopy E-Videos https://eref.thieme.de/e-videos
*E-Videos* is an open access online section of the journal *Endoscopy* , reporting on interesting cases and new techniques in gastroenterological endoscopy. All papers include a high-quality video and are published with a Creative Commons CC-BY license. Endoscopy E-Videos qualify for HINARI discounts and waivers and eligibility is automatically checked during the submission process. We grant 100% waivers to articles whose corresponding authors are based in Group A countries and 50% waivers to those who are based in Group B countries as classified by Research4Life (see: https://www.research4life.org/access/eligibility/ ). This section has its own submission website at https://mc.manuscriptcentral.com/e-videos .
